# First patient management of COVID-19 in Changsha, China: a case report

**DOI:** 10.1186/s12879-020-05545-y

**Published:** 2020-11-11

**Authors:** Jian Zhou, Ziqin Cao, Wanchun Wang, Kang Huang, Fang Zheng, Yuanlin Xie, Dixuan Jiang, Zhiguo Zhou

**Affiliations:** 1 Department of Orthopedics, The Second Xiangya Hospital, Central South University, Changsha, 410011 Hunan China; 2 Department of Respiratory Medicine, The First Hospital of Changsha City, No.311 Yingpan Road, Kaifu District, Changsha, 410005 Hunan Province China

**Keywords:** Cured patient, COVID-19, China, Case report

## Abstract

**Background:**

In December 2019, the novel coronavirus disease 2019 (COVID-19) emerged in Wuhan, Hubei Province, China. It rapidly spread and many cases were identified in multiple countries, posing a global health problem. Here, we report the first patient cured of COVID-19 infection in Changsha, China, and the symptoms, diagnosis, treatment, and management of this patient are all described in this report.

**Case presentation:**

A 57-year-old woman developed cough and fever after returning to Changsha from Wuhan on January 9, 2020. She tested positive for COVID-19 infection, a diagnosis which was supported by chest CT. The patient was treated with lopinavir and ritonavir tablets and interferon alfa-2b injection. A low dose of glucocorticoids was used for a short period to control bilateral lung immune response, and this patient avoided being crushed by cytokine storms that might have occurred. The clinical condition of this patient improved, and a COVID-19 assay conducted on January 25, 2020 generated negative results. This patient recovered and was discharged on January 30, 2020.

**Conclusions:**

Currently, there are numerous reports on COVID-19 infections focusing on the disease’s epidemiological and clinical characteristics. This case describes the symptoms, diagnosis, treatment, and management of a patient cured of COVID-19 infection, which may serve as reference for future cases, while further studies are needed.

## Background

Coronavirus disease 2019 (COVID-19) infections can lead to severe respiratory disease. At the beginning of December 2019, a cluster of patients with COVID-19 infection was reported in China [1, 2, 3]. A previous report on COVID-19 indicated that human-to-human transmission of COVID-19 had been occurring [4]. By August 31, 2020, a cumulative total of nearly 25 million cases and 800,000 deaths have been reported [5]. COVID-19’sepidemiological clinical features were reported in several studies [4, 6, 7]. The first case of COVID-19 infection in the United States was reported on January 20, 2020, and the clinical condition of this patient apparently improved following treatment with intravenous remdesivir [8]. In this report, we describe the symptoms, diagnosis, treatment, and management of the first patient cured of COVID-19 infection in Changsha, China.

## Case presentation

**Fig. 1:**
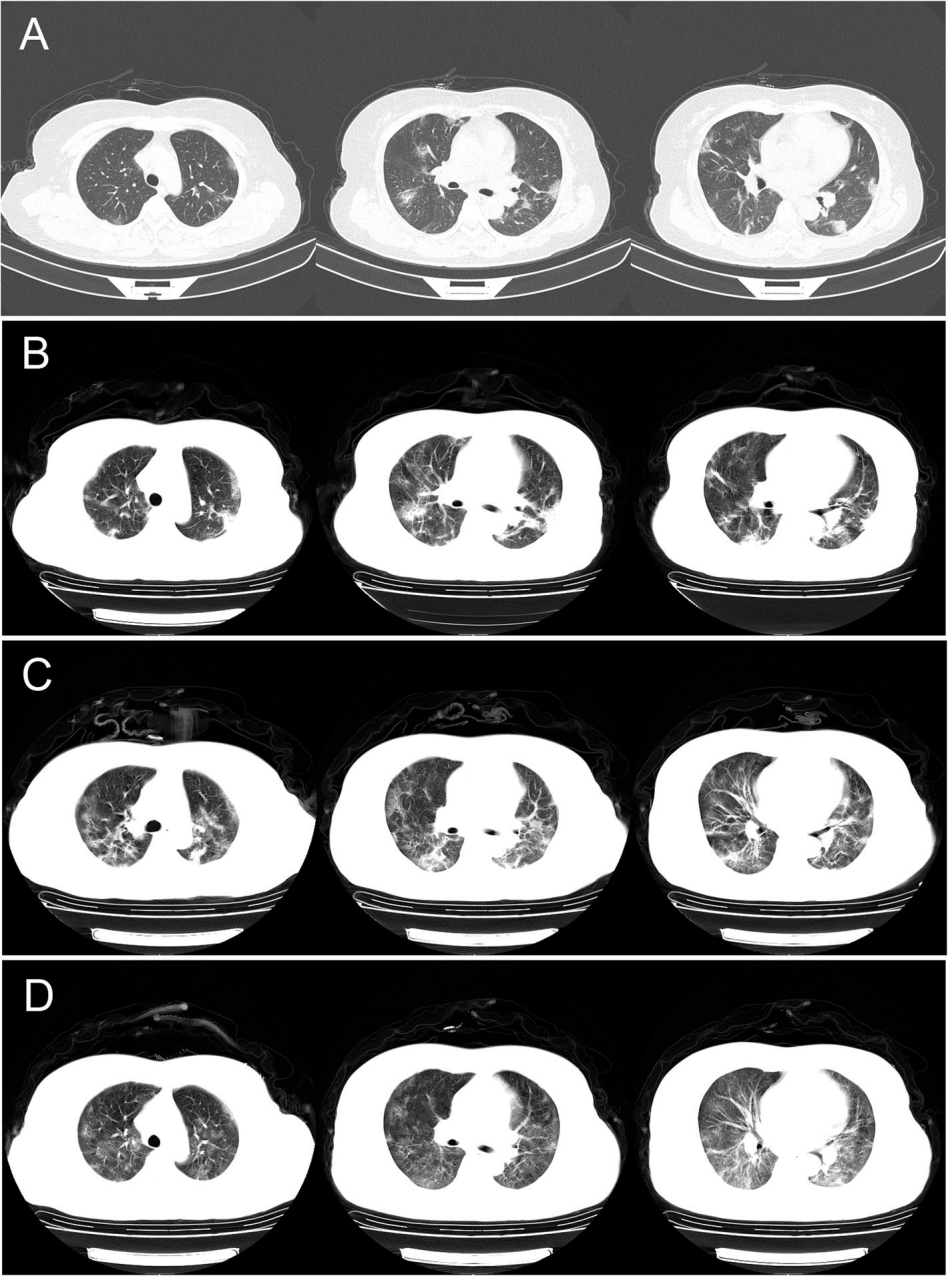
Chest CT of this patient. **a** Chest CT was obtained on January 16, 2020 (hospital day 1, illness day 7). Multiple patchy shadows and cord-like ground-glass opacity (GGO) under the pleura and bilateral lungs were observed. **b** Chest CT was obtained on January 19, 2020 (hospital day 4, illness day 10). The texture of the trachea and blood vessels in both lungs showed thickening. GGO increased, and the original GGO was consolidated. **c** Chest CT was obtained on January 23, 2020 (hospital day 8, illness day 14). The patchy lesions in both lungs were absorbed, and the fiber shadow increased in size. **d** Chest CT was obtained on January 30, 2020 (hospital day 15, illness day 21). The consolidation in the bilateral lungs was further absorbed. The fiber strands were reduced, and GGO increased slightly

On January 16, 2020, a 57-year-old woman came to the emergency department of the First Hospital of Changsha City, China. After 10 min, she was taken into the examination room and evaluated by the emergency physician. The chief complaints of this patient were cough and fever, with general weakness and muscle aches that developed after returning to Changsha from Wuhan 7 days prior. Given her symptoms and recent travel history, she decided to see a health care provider. The patient had a history of hypertension, carotid plaque, hypothyroidism, and chronic gastritis, without a smoking or drinking habit. The physical examination revealed the following: body temperature: 38.0 °C, blood pressure: 143/76 mmHg, pulse: 78 beats per minute, respiratory rate: 13 breaths per minute, and oxygen saturation: 96%. She had throat congestion and thick bilateral breath sounds in the lungs. To evaluate the abnormal breath sounds, we performed a chest CT examination, which revealed bilateral pneumonia (Fig. 1a). The results of nucleic acid amplification testing (NAAT) for influenza A and B were negative. Given the patient’s travel history and CT findings, Hunan Province and Chinese Center for Disease Control and Prevention (CCDC) were immediately notified. CCDC staff required us to test the patient for COVID-19, even though the patient disclosed that she had never been to the Huanan seafood market and reported no known contact with ill persons in the past month. Specimens were collected following CCDC guidance. After specimen collection, she was admitted to the isolation ward of the First Hospital of Changsha City. On admission, the patient reported persistent dry cough, fatigue, headache, sore throat, and chest pain for a week. Upon physical examination, the patient was found to have throat congestion without other remarkable findings.

**Fig. 2:**
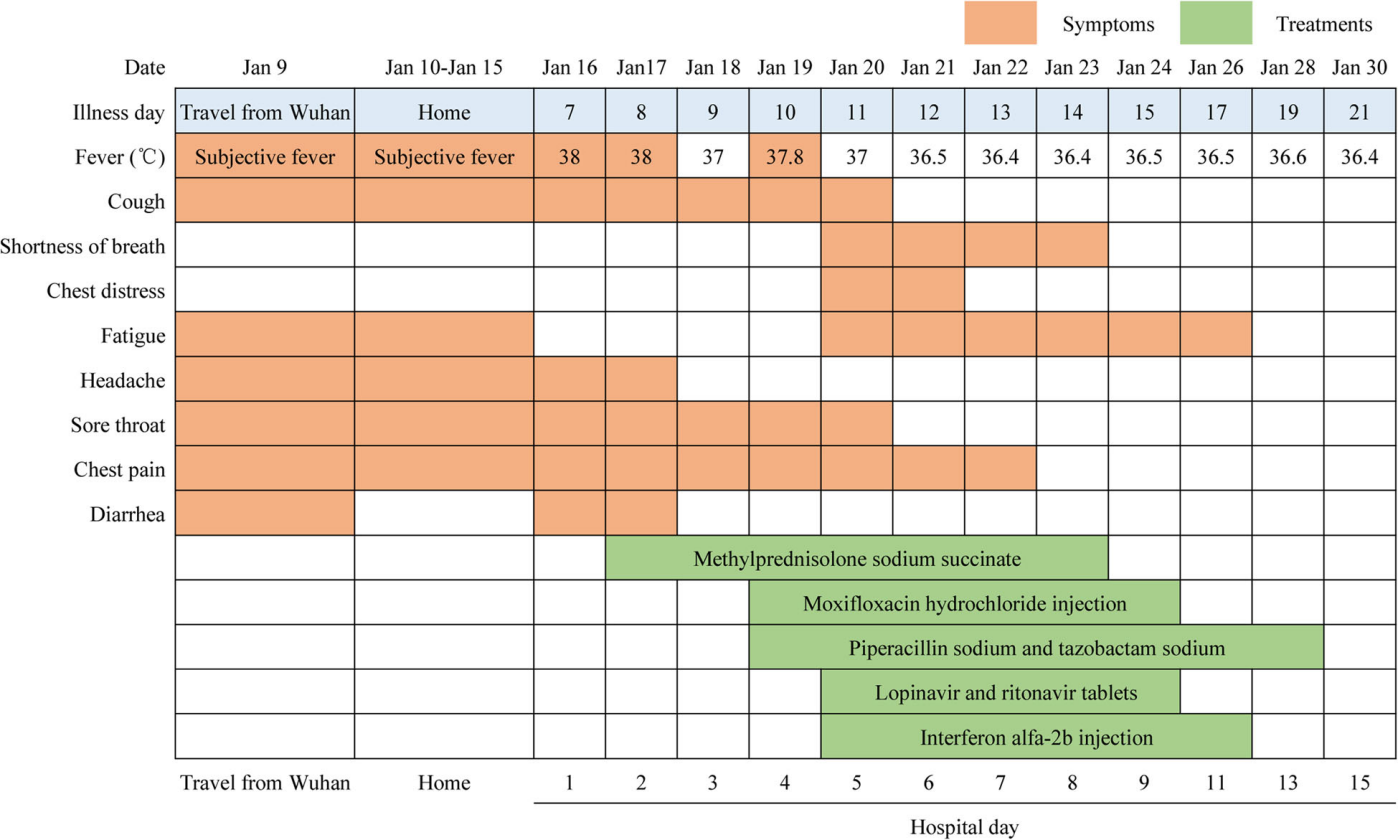
Clinical symptoms and treatments from January 9 to January 30, 2020

On hospital days 2–4 (illness days 8–10), the patient’s vital signs remained largely stable. She reported that her cough and sore throat were worse than before, accompanied by chest pain and a small amount of sputum. Intermittent fever and sore throat were still reported (Fig. 2). Supportive treatment was performed at this stage, and methylprednisolone sodium succinate 40 mg QD intravenously was given to inhibit lung inflammation. During this period, we found that the patient developed melena in the morning, indicating that we should be aware of the possibility of upper gastrointestinal bleeding. The patient was treated with pantoprazole for acid suppression. Ambroxol (30 mg BID intravenously), limonene, and pinene enteric soft capsules (0.3 g TID peros) were used to expel sputum. Laboratory results on hospital days 1–3 (illness days 7–9) reflected leukopenia, neutropenia, lymphopenia, and reduced hematocrit. Additionally, elevated levels of lactate dehydrogenase and C-reactive protein were observed (Table 1). According to the suggestion of *The Diagnosis and Treatment of Pneumonitis with COVID-19 Infection* (DTPI) published by National Health Commission of the PRC, we generally monitor the patient’s blood oxygen saturation and oxygenation index closely. When the patient’s blood oxygen saturation is below 93%, respiratory support is given. This patient has not reached the point where tracheal intubation is required, so respiratory support was not given to this patient.

**Table 1:** Clinical Laboratory Results

Measure	Reference range	16	17	18	19	20	21	22	23	24	26	29	30
White-cell count (10^9^/L)	4–10	2.45^a^	2.29^a^	2.2^a^	6.15	8.56	9.73	9.33	4.82	4.3	4.21	6.88	6.12
Red-cell count (10^12^/L)	3.5–5.5	3.96	3.8	4.4	3.96	4.11	4.1	4.18	3.99	4.07	4.1	3.43^a^	3.6
Absolute neutrophil count (10^9^/L)	2–7	1.75^a^	1.44^a^	1.45^a^	5.34	7.97^b^	9.21^b^	8.59^b^	4.08	2.92	2.32	4.7	3.86
Absolute lymphocyte count (10^9^/L)	0.8–4	0.6^a^	0.73^a^	0.69^a^	0.62^a^	0.45^a^	0.32^a^	0.43^a^	0.49^a^	0.92^a^	1.39^a^	1.48^a^	1.6^a^
Platelet count (10^9^/L)	100–300	145	139	181	203	231	290	326^b^	326^b^	358^b^	342^b^	325^b^	358^b^
Hemoglobin (g/L)	110–160	110	106^a^	122	108^a^	114	113	114	110	111	111	94^a^	100^a^
Hematocrit (%)	37–54	34.4^a^	32.8^a^	38.1	34.1^a^	35.1^a^	35.1^a^	35.8^a^	34^a^	35.1^a^	35.6^a^	30.1^a^	31.4^a^
Potassium (mmol/L)	3.5–5.5	4.09	–	3.71	3.7	3.3^a^	4.06	3.69	3.77	4.12	3.72	4.08	4.12
Sodium (mmol/L)	133–149	134.4	–	135.2	134	136.3	134.7	139.5	137.7	134.2	135.8	135.8	135.6
Chloride (mmol/L)	95–110	99.8	–	101.9	101	101.6	102.3	101.9	103.2	100	102.1	102.5	103.8
Calcium (mmol/L)	1.05–1.35	–	–	1.19	1.21	1.19	1.18	1.21	1.22	1.19	1.2	1.25	1.2
Alanine aminotransferase (U/L)	0–42	16.2	20.6	23.88	21.84	21.31	18.94	26.12	21.81	16.74	22.37	19.54	19.18
Aspartate aminotransferase (U/L)	0–37	35.6	43.4^b^	37.4^b^	27.47	22.7	19.44	17.51	16.3	11.27	15.91	14.04	18.88
Total bilirubin (μmol/L)	3.4–20.5	5.2	4.6	5.94	5.3	6.23	7.83	13.41	13.54	10.98	5.32	5.43	5.03
Total protein (g/L)	60–83	58.9^a^	58.5^a^	63.17	56^a^	59.3^a^	67.79	64.42	58.2^a^	58.51^a^	57.99^a^	55.43^a^	62.03
Albumin (g/L)	35–55	35.4	35.8	37.6	33.51^a^	33.38^a^	34.56^a^	32.88^a^	28.08^a^	28.73^a^	34.33^a^	31.67^a^	34.79^a^
Blood urea nitrogen	2.86–8.2	2.5^a^	2.47^a^	3.85	4.32	4.61	5.99	13.29^b^	10.29^b^	8.05	6.63	5.45	4.21
Creatinine (μmol/L)	19.8–87.1	63.8	65.6	49.85	52.44	51.73	55.2	98.25^b^	77.66	82.63	52.08	48.85	57.25
Uric acid (μmol/L)	149–430	247	196	236.6	206.6	192.9	196	343.6	188.8	125.4^a^	88.1^a^	135.2^a^	116.7^a^
Creatine kinase (U/L)	10–190	160.3	–	149.2	96.7	60	43	19.2	16.6	14.7	19.1	26	34.1
Creatine kinase-MB (U/L)	0–24	35.2^b^	17.2	19.4	18.2	17.8	28.6^b^	6.9	12.8	5.4	3.9	5.4	4.7
Lactate dehydrogenase (U/L)	80–245	306^b^	282^b^	286.3^b^	275.9^b^	280.1^b^	266.4^b^	218.9	176.8	150.8	128.6	139.9	168.5
Venous lactate (mg/L)	120–160	–	–	–	–	705.2^b^	295.8^b^	831.3^b^	902.3^b^	812^b^	838.4^b^	880.1^b^	803.7^b^
C-reactive protein (mg/L)	0–8	19.7^b^	18.8^b^	17.05^b^	16.07^b^	55.42^b^	43.19^b^	22.71^b^	12.61^b^	6.49	3.72	–	10.37^b^
Partial pressure of carbon dioxide (mmHg)	35–45	31.8^a^	–	24.8^a^	28.1^a^	36.1	27.5^a^	39.4	38.7	43.1	38.8	33.5^a^	27.1^a^
Partial pressure of oxygen (mmHg)	80–100	78.8^a^	–	119.2^b^	116.8^b^	56.8^a^	78.2^a^	77.2^a^	61.9^a^	97.9	74.6^a^	89.5	102.5^b^
PaO_2_/FiO_2_ (mmHg)	400–500	272^a^	–	411	403	172^a^	270^a^	234^a^	188^a^	316^a^	241^a^	309^a^	353^a^
Blood oxygen saturation (%)	91.9–99.9	96.6	–	98.6	98.6	92	96.5	96.4	93.3	97.7	95.8	97.6	98.2
Carbon dioxide (mmol/L)	24–32	–	–	18.6^a^	21.9^a^	28.6	21.1^a^	31.5	29.3	31.5	29.2	27.5	22.2^a^
Procalcitonin (ng/ml)	0–0.05	< 0.05	< 0.05	< 0.05	< 0.05	< 0.05	< 0.05	< 0.05	< 0.05	< 0.05	< 0.05	< 0.05	< 0.05
D-Dimer (μg/ml)	0–1	0.28	0.31	0.35	0.38	0.47	0.63	0.61	0.66	0.54	0.43	0.45	0.55

^a^The value in the patient was below normal

^b^The value in the patient was above normal

On hospital day 4 (illness day 10), re-examination of the lung CT showed that lung inflammation had progressed (Fig. 1b). The dose of methylprednisolone sodium succinate was changed to 40 mg Q12H intravenously, and intravenous human immunoglobulin (Ph4) 5 g BID was added to inhibit lung inflammation. Given the clinical presentation, treatment with piperacillin sodium and tazobactam sodium (4.5 g Q8H intravenously) and moxifloxacin hydrochloride and sodium chloride injection (0.4 g QD intravenously) were initiated.

On hospital day 5 (illness day 11), the CCDC confirmed that the oropharyngeal swabs of this patient tested positive for COVID-19 by real-time reverse transcriptase-polymerase chain reaction (rRT-PCR) assay. According to the suggestion of *The Diagnosis and Treatment of Pneumonitis with COVID-19 Infection* (DTPI) published by the National Health Commission of the PRC, lopinavir and ritonavir tablets (2 pills BID by mouth), which had been used to treat HIV infection in the past, as well as interferon alfa-2b injection (5 million IU added into 2 mL of sterile water, inhalation BID) were added to the patient’s treatment regimen. On hospital day 8 (illness day 14), the temperature of this patient dropped to 36.4 °C. Moreover, her appetite improved, and she was asymptomatic apart from fatigue and chest pain. A comparison of a new CT scan and the previous CT images showed that the bilateral patchy lesions in her lungs had been absorbed (Fig. 1c). Methylprednisolone sodium succinate was then discontinued. On hospital day 9 (illness day 15), the blood pressure of this patient dropped to 85/55 mmHg. Therefore, Shenmai injection 50 mg QD intravenously was used, and the patient’s blood pressure rose to 113/70 mmHg. On hospital day 10 (illness day 16), a negative result was obtained for the COVID-19 assay. This patient reported that her cough and fever had abated and her clinical condition improved. Lopinavir and ritonavir were then discontinued on hospital day 10 (illness day 16). Interferon alfa-2b injection and antibiotics were discontinued on hospital day 11 (illness day 17). On hospital day 14 (illness day 20), this patient again tested negative for COVID-19 by rRT-PCR assay, and was discharged on January 30, 2020 (hospital day 15, illness day 21).

## Methods

### Diagnostic process

In accordance with the DTPI guidelines, this case was diagnosed based on epidemiological history and clinical manifestations:

1. Epidemiological history (must comply with any one of the following)
  1. Travel or residential history in Wuhan, China within 14 days before the onset of illness;
  2. Exposure to patients with fever or respiratory symptoms from Wuhan City within 14 days before the onset of illness;
  3. Aggregative onset or epidemiological association with new coronavirus infection.
2. Clinical manifestations (must comply with any two of the following)
  1. Fever (> 37.3 °C);
  2. Imaging characteristics of pneumonia;
  3. Normal or decreased total number of white blood cells, or decreased lymphocyte count in the early stage of onset.
3. Laboratory testing
  1. Testing positive for COVID-19 via rRT-PCR;
  2. The results of sequencing using respiratory specimens or blood specimens are highly homologous with COVID-19.

### Laboratory testing

The COVID-19 laboratory test assays were conducted according to WHO recommendations [9]. Laboratory identification of COVID-19 was performed by three different institutions: the First Hospital of Changsha City, Hunan CDC, and CCDC. Upper and lower respiratory tract specimens were obtained from this patient thrice (hospital days 3, 8, and 13). RNA was obtained and further tested by rRT-PCR through the same method described in a previous study [2]. This study also tested for other respiratory viruses (influenza A virus, influenza B virus, and respiratory syncytial virus) and parainfluenza virus. In addition, this patient underwent chest X-rays and chest CT.

## Results

CT imaging showed multiple patchy shadows and cord-like ground-glass opacity (GGO) under the pleura and bilateral lungs (hospital day 1, illness day 7, Fig. 1a). Furthermore, a second chest CT indicated that the texture of the trachea and blood vessels in both lungs had thickened. GGO increased, and the original GGO was consolidated (hospital day 4, illness day 10, Fig. 1b). Oropharyngeal swabs were obtained from this patient on hospital days 3, 8, and 13. A positive result for COVID-19 was obtained on hospital day 5. On hospital day 8 (illness day 14), 3 days after beginning treatment with lopinavir and ritonavir tablets combined with interferon alfa-2b injection, a comparison of a third chest CT with the previous CT images obtained on hospital day 4 indicated that the patchy lesions in the bilateral lungs were absorbed, and the fiber shadow was increased (Fig. 1c). On hospital day 10 (illness day 16), rRT-PCR assay was performed to test for active COVID-10 infection, and a negative result was obtained. On hospital day 15 (illness day 21), a fourth CT showed that the consolidation in the bilateral lungs had been absorbed to a greater extent. The fiber strands were reduced, and GGO increased slightly (Fig. 1d). The COVID-19 infection of this case was checked again, and a negative result was obtained.

### Discussion and conclusions

Currently, the full spectrum and transmission dynamics of these infections are unclear. Here, we describe a patient cured of COVID-19 in Changsha, China. During the progression of the patient’s disease, a low dose of glucocorticoids was used to control the immune response, and the patient avoided being crushed by cytokine storms that might have occurred, thereby reducing the risk of complications such as acute respiratory distress syndrome (ARDS). During the severe acute respiratory syndrome (SARS) period in 2003, the use of high doses of glucocorticoids caused a series of sequelae in survivors, such as osteonecrosis of the femoral head and glucose metabolism disorders. In view of this lesson, we administered a small dose (40–80 mg/day) of glucocorticoids only within a short period (5 days) and adjusted the dose and time of the medication according to the dynamic changes shown on the patient’s chest CT imaging, which achieved good results.

Our patient reported that she returned to Changsha from Wuhan without visiting the Huanan seafood market. A previous study revealed that COVID-19 had been spreading via person-to-person transmission [10]. As of February 4, 2020, no secondary cases of COVID-19 related to this patient were confirmed. Currently, the clinical spectrum of COVID-19 infections is limited. Several reports have described various complications related to COVID-19 [2, 6, 11]. In this study, this patient initially manifested fever and cough with eukopenia, neutropenia, and lymphopenia. The report on the first case of COVID-19 in the United States indicated no sign of pneumonia on the patient’s chest X-ray on illness day 4, which indicated that this disease may have a latency period. The nonspecific signs of COVID-19 infection may be clinically different from those of other infectious diseases. Many hospitals in China currently use lopinavir and ritonavir tablets combined with interferon alfa-2b injection to treat COVID-19. In this report, this patient was also treated with the above-mentioned medication regimen. However, additional studies are needed to confirm the effect of this therapeutic schedule. A multicenter randomized controlled trial on the treatment of COVID-19 using lopinavir and ritonavir tablets is currently in progress in China.

The present study emphasizes the need to confirm the full spectrum and pathogenesis related to COVID-19 infections. Additional information about this disease is necessary for its clinical management. We should make every possible effort to control this infectious disease.

## Data Availability

The datasets used and/or analyzed during the current study are available from the corresponding author upon reasonable request.s.

## Abbreviations

COVID-19: Coronavirus disease 2019
GGO: Ground-glass opacity
CCDC: Chinese Center for Disease Control and Prevention
NAAT: Nucleic acid amplification test
rRT-PCR: Real-time reverse transcriptase-polymerase chain reaction
DTPI: *The Diagnosis and Treatment of Pneumonitis with COVID-19 Infection*
ARDS: Acute respiratory distress syndrome
SARS: Severe acute respiratory syndrome

## References

[1] World Health Organization (2020). Pneumonia of unknown cause - China.

[2] Zhu N, Zhang D, Wang W, Li X, Yang B, Song J, Zhao X, Huang B, Shi W, Lu R, et al. A Novel Coronavirus from Patients with Pneumonia in China, 2019. N Engl J Med. 2020;382(8):727-33. 10.1056/NEJMoa2001017 PMC7092803 31978945

[3] Centers for Disease Control and Prevention (2020). 2019 Novel coronavirus, Wuhan, China: 2019-nCoV situation summary.

[4] Li Q, Guan X, Wu P, Wang X, Zhou L, Tong Y, Ren R, Leung K, Lau E, Wong JY, et al. Early transmission dynamics in Wuhan, China, of novel coronavirus-infected pneumonia. N Engl J Med. 2020;382(13):1199-207. 10.1056/NEJMoa2001316 PMC7121484 31995857

[5] World Health Organization (2020). Novel Coronavirus (2019-nCoV) situation reports. Weekly Epidemiological Update Coronavirus disease 2019 (COVID-19).

[6] Huang C, Wang Y, Li X, Ren L, Zhao J, Hu Y, Zhang L, Fan G, Xu J, Gu X, et al. Clinical features of patients infected with 2019 novel coronavirus in Wuhan, China. Lancet. 2020;395(10223):497-506. 10.1016/S0140-6736(20)30183-5 PMC7159299 31986264

[7] Chen N, Zhou M, Dong X, Qu J, Gong F, Han Y, Qiu Y, Wang J, Liu Y, Wei Y, et al. Epidemiological and clinical characteristics of 99 cases of 2019 novel coronavirus pneumonia in Wuhan, China: a descriptive study. Lancet. 2020;395(10223):507-1. 10.1016/S0140-6736(20)30211-7 PMC7135076 32007143

[8] Holshue ML, DeBolt C, Lindquist S, Lofy KH, Wiesman J, Bruce H, Spitters C, Ericson K, Wilkerson S, Tural A, et al. First case of 2019 novel coronavirus in the United States. N Engl J Med. 2020;382(10):929-36. 10.1056/NEJMoa2001191 PMC7092802 32004427

[9] WHO (2020). Laboratory diagnostics for novel coronavirus.

[10] Chan JF, Yuan S, Kok KH, To KK, Chu H, Yang J, Xing F, Liu J, Yip CC, Poon RW, et al. A familial cluster of pneumonia associated with the 2019 novel coronavirus indicating person-to-person transmission: a study of a family cluster. Lancet. 2020;395(10223):514-23. 10.1016/S0140-6736(20)30154-9 PMC7159286 31986261

[11] Centers for Disease Control and Prevention (2020). Interim guidelines for collecting, handling, and testing clinical specimens from patients under investigation (PUIs) for 2019 novel coronavirus (2019-nCoV).

